# The chaperone Tsr2 regulates Rps26 release and reincorporation from mature ribosomes to enable a reversible, ribosome-mediated response to stress

**DOI:** 10.1126/sciadv.abl4386

**Published:** 2022-02-25

**Authors:** Yoon-Mo Yang, Katrin Karbstein

**Affiliations:** 1 Department of Integrative Structural and Computational Biology, The Scripps Research Institute, Jupiter, FL 33458, USA.; 2 HHMI Faculty Scholar, Chevy Chase, MD 20815, USA.

## Abstract

Although ribosome assembly is quality controlled to maintain protein homeostasis, different ribosome populations have been described. How these form, especially under stress conditions that affect energy levels and stop the energy-intensive production of ribosomes, remains unknown. Here, we demonstrate how a physiologically relevant ribosome population arises during high Na^+^, sorbitol, or pH stress via dissociation of Rps26 from fully assembled ribosomes to enable a translational response to these stresses. The chaperone Tsr2 releases Rps26 in the presence of high Na^+^ or pH in vitro and is required for Rps26 release in vivo. Moreover, Tsr2 stores free Rps26 and promotes reincorporation of the protein, thereby repairing the subunit after the Na^+^ stress subsides. Our data implicate a residue in Rps26 involved in Diamond Blackfan Anemia in mediating the effects of Na^+^. These data demonstrate how different ribosome populations can arise rapidly, without major energy input and without bypass of quality control mechanisms.

## Teaser

Chaperone-mediated release and reincorporation of proteins from ribosomes enable a physiological translational stress response.

## INTRODUCTION

Ribosomes synthesize proteins in all living organisms. Well over half of all transcription and translation events are dedicated to the synthesis of the 1000 to 2000 ribosomes/min, which are required to maintain ribosome populations (*1*). This task is further complicated by the need to ensure that ribosomes are correctly assembled. For that purpose, cells have co-opted the translational machinery to establish a quality control pathway, which test-drives nascent ribosomes in a translation-like cycle (*2*, *3*) and establishes checkpoints to prevent immature or misassembled ribosomes from entering the translating pool (*4*–*6*). The importance of producing a homogeneous population of correctly assembled ribosomes is supported not only by these findings and the observation that mutations that bypass quality control occur in cancer cells (*5*–*7*) but also from findings that cancer cells accumulate ribosomes with altered protein stoichiometry (*8*–*11*). Moreover, insufficiency of ribosomal proteins can lead to the accumulation of ribosomes lacking these proteins (*12*, *13*) and, in human cells, predisposes to cancer (*10*, *14*).

At the same time, recent work has provided ample evidence for subpopulations of ribosomes that differ in composition (*15*–*19*). While many of these are associated with disease states and arise from haploinsufficiency of ribosomal proteins (*20*–*22*), others are found in wild-type (wt) cells (*12*, *23*–*25*). However, whether these have functional relevance or are potentially even degradation intermediates is unclear in most cases (*19*).

While ribosomes are costly to assemble, they are exceptionally stable and not turned over during the division cycle of most cells (*1*, *26*). In the case of ribosomes deposited into the egg during oogenesis, these ribosomes must persist throughout the fertile life of the animal, which, in the case of humans, can be decades. Whether ribosomes become damaged over these extended time periods and then repaired remains unknown. Therefore, in addition to being functional, or represent nonfunctional degradation intermediates, ribosomes lacking individual ribosomal proteins could also represent intermediates of yet-to-be-found repair pathways.

A ribosome subpopulation with a known physiological role are Rps26-deficient ribosomes (*12*). Formed when yeast cells are exposed to high Na^+^ concentrations or high pH, they enable the translation of mRNAs containing an otherwise unfavorable guanosine residue at the −4 position of the Kozak sequence. This loss of preference toward the canonical −4A is explained by Rps26 interaction with this residue. About one-quarter of all mRNAs in yeast are differentially bound by Rps26-deficient ribosomes, including those enabling the biological response to high Na^+^ and high pH (*12*). How these Rps26-deficient ribosomes are produced in cells is not known. In particular, it is unclear whether they are newly made, and evading quality control mechanisms to ensure all proteins are incorporated, or if instead they are produced by release of Rps26 from preexisting completely assembled ribosomes.

Using these physiological Rps26-deficient ribosomes as a case study, we report here how mature ribosomes can be remodeled under high-Na^+^ conditions and repaired once the stress subsides. Pulse-chase experiments demonstrate that Rps26-deficient ribosomes are formed by release of Rps26 from preexisting, fully matured ribosomes, rather than by bypassing Rps26 incorporation into newly made ribosomes. Furthermore, we show that Rps26 is reincorporated once the Na^+^ is removed, demonstrating active repair of these ribosomes. Our experiments suggest that binding of metal ions and protons to specific sites at the Rps26-RNA interface allows for direct sensing of Na^+^ and H^+^ concentrations, and implicate Asp33, a residue mutated in Diamond Blackfan Anemia (DBA) in mediating salt-dependent effects. In vivo and in vitro experiments show that Rps26 release is enabled by the chaperone Tsr2, which has been previously suggested to deliver Rps26 to ribosomes (*27*, *28*). We show that Tsr2 promotes Rps26 dissociation and stores the released protein for reincorporation. This observation supports the importance of Tsr2 in adjusting the proper ratio of Rps26-containing and deficient ribosomes.

## RESULTS

### Rps26-deficient ribosomes are generated from preexisting mature ribosomes under stress

We have previously shown that Rps26-deficient ribosomes accumulate in yeast cells exposed to high Na^+^ concentrations or pH (*12*). Because Rps26-deficient ribosomes no longer preferentially recognize mRNAs with an adenosine in the −4 position, to support their preferential translation, accumulation of Rps26 ribosomes decreases the translation of these otherwise well-translated mRNAs, allowing the translation of mRNAs with a −4G, which are otherwise discriminated against (*12*). mRNAs in the Rim101 pathway, responding to high pH, and the Hog1 pathway, responding to high osmolarity, are enriched on Rps26-deficient ribosomes (*12*), and correspondingly, Rps26-deficient yeast are resistant to high Na^+^ concentrations or pH (*12*). However, the Hog1 pathway is also efficiently induced by high concentrations of sorbitol (*29*). Thus, we wanted to test whether Rps26 deficiency would also lead to sorbitol resistance, as predicted. As before (*12*), we used a yeast strain where endogenous Rps26 could be depleted by growth in glucose and supplemented this strain with plasmids encoding Rps26, either under the constitutive strong TEF2 promoter or the doxycycline (dox)–repressible TET promoter. Addition of 1 M NaCl or 1 M sorbitol produced growth defects, although these defects were much stronger with NaCl than sorbitol (fig. S1A). Addition to NaCl or sorbitol improves the growth of yeast cells where Rps26 is depleted by dox addition. Thus, yeast depleted for Rps26 are resistant not only to NaCl, as previously shown, but also to sorbitol (fig. S1B). The common growth rate for Rps26-depleted cells in NaCl and sorbitol likely reflects a bottleneck due to reduced 40*S* ribosome concentrations.

**Fig. 1.**
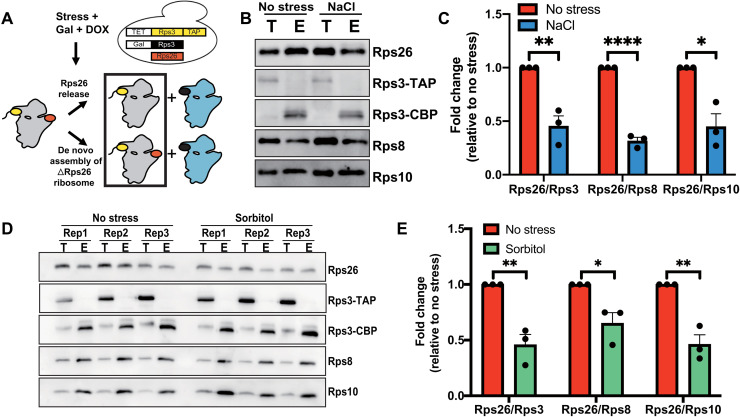
Rps26-deficient ribosomes arise from preexisting 40*S* subunits. (**A**) Pulse-chase experiments to separate preexisting ribosomes (gray) from newly made ribosomes (blue) rely on a yeast strain with Rps3-TAP produced from a TET-repressible promoter (yellow), and Rps3 (black) produced from a galactose-inducible/glucose-repressible promoter. Rps26 is shown in red. By shifting this strain from glucose to galactose/dox, preexisting ribosomes are marked with the Rps3-TAP affinity purification handle and will be in the TAP-elution, as indicated by the black box. (**B**) Western blot of preexisting (Rps3-TAP) ribosomes isolated by affinity purification from cells treated or not treated with 1 M NaCl before lysis. T, total lysate; E, elution. Note that elution from the IgG beads by TEV protease converts Rps3-TAP (Rps3–CBP–protein A) to Rps3-CBP (Rps3-calmodulin–binding protein). (**C**) Quantification of data in (B). Data are averaged from three biological replicates. Error bars represent the SEM, and significance was determined using an unpaired *t* test. *P < 0.05; **P < 0.01; ****P < 0.0001. (**D**) Western blot of preexisting (Rps3-TAP) ribosomes isolated by affinity purification from cells treated or not treated with 1 M sorbitol before lysis. (**E**) Quantification of data in (D). Data are averages from three biological replicates. Error bars represent the SEM, and significance was determined using an unpaired *t* test. *P < 0.05; **P < 0.01.

Next, we asked whether the Rps26-deficient ribosomes that are formed in yeast exposed to stress arise by release of Rps26 from preexisting mature ribosomes or by omission of Rps26 during assembly of new ribosomes (Fig. 1A). To distinguish between these pathways, we designed a pulse-chase experiment to differentiate between premade and newly made ribosomes (Fig. 1A). In this experiment, preexisting ribosomes are marked with tandem-affinity-purification (TAP)–tagged Rps3 whose expression relies on the dox-repressible TET promoter. Untagged Rps3 is under galactose-inducible/glucose-repressible control. Cells were initially grown in glucose media, such that all preexisting ribosomes will be marked with Rps3-TAP. In mid-log phase, cells were switched to galactose media–containing dox, such that newly made ribosomes contain untagged Rps3. Control experiments confirm the rapid up-regulation of Rps3 and down-regulation of Rps3-TAP mRNAs under these conditions (fig. S1, C and D). NaCl was added when the cells are switched to galactose/dox media. Thus, ribosomes made before the stress (preexisting ribosomes) are marked with Rps3-TAP, while newly made ribosomes are untagged. These two ribosome populations were separated using immunoglobulin G (IgG) beads, and the level of Rps26 in the preexisting, TAP-tagged ribosomes (elution) was measured by Western blotting in comparison with three other ribosomal proteins (Rps3, Rps8, and Rps10) as previously described (*12*). Rps26 levels in preexisting ribosomes (elution) were decreased approximately 50% (Fig. 1, B and C) when yeast cells were exposed to NaCl stress. Similarly, when yeast are exposed to sorbitol, and preexisting ribosomes are similarly tagged and purified via IgG beads, the Rps26 in the preexisting ribosomes (elution) was reduced (Fig. 1, D and E). These observations demonstrate that Rps26-deficient ribosomes arise from release of Rps26 from preexisting ribosomes upon exposure to high Na^+^ or sorbitol stress. This is consistent with our previous observation that in high Na^+^, Rps26 mRNA levels were down-regulated as much, but not more than, other tested mRNAs (*12*), and the finding that stress conditions block ribosome assembly (*1*, *30*), which would also block the de novo formation of Rps26-deficient ribosomes.

### Tsr2 dissociates Rps26 from mature ribosomes in vitro

**Fig. 2.**
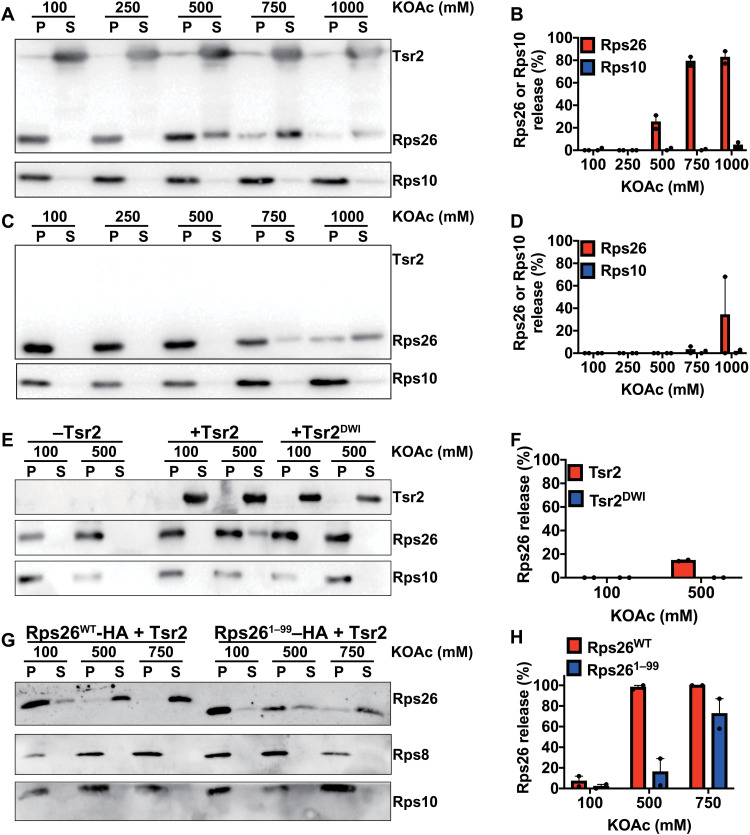
Tsr2 promotes Rps26 release from mature 40*S* subunits in the presence of high salt in vitro. Western blot analysis of pelleted (P) ribosomes and released proteins in the supernatant (S). Mature 40*S* subunits purified from yeast were incubated with (**A**) or without (**C**) recombinant Tsr2 under different salt concentrations. (**B** and **D**) Released Rps26 or Rps10 was quantified from (A) and (C) with two independent replicates for each condition. Error bars represent the SEM. (**E**) Rps26 release assay with the Rps26 interaction–deficient Tsr2_DWI mutant. (**F**) Quantification of Rps26 in (E) with two independent replicates for each condition. Error bars represent the SEM. (**G**) Rps26 release assay with the Tsr2 interaction–deficient Rps26^1–99^ (lacking residues 100 to 119) truncation mutant. (**H**) Quantification of released Rps26 in (G) with two independent replicates for each condition. Error bars represent the SEM.

The data above demonstrate that Rps26 is released from preexisting mature ribosomes to yield Rps26-deficient ribosomes under high Na^+^ (or sorbitol) stress. This led us to ask next how Rps26 dissociation occurs within cells. Tsr2 is a chaperone for Rps26, which stabilizes the protein outside of the ribosome (*27*, *28*, *31*). A role for Tsr2 in Rps26 incorporation into 40*S* subunits has also been suggested (*27*, *28*). Given Tsr2’s ability to bind and stabilize Rps26, we wondered if it could also release Rps26 from mature 40*S*. To test this hypothesis, we developed an in vitro release assay. After incubation of purified 40*S* subunits with purified recombinant Tsr2 in varying salt concentrations, the samples were loaded onto a sucrose cushion and spun at high speed to pellet ribosomes (and its bound Rps26), thereby separating it from released Tsr2-bound Rps26, which will remain in the supernatant. The data in Fig. 2 (A and B) demonstrate that at increased concentrations of KOAc, Tsr2 releases Rps26 from mature 40*S* subunits. Release from the 40*S* subunit was specific for Rps26, as other ribosomal proteins were observed in the pellet fraction (Fig. 2A and fig. S2, A and B). Last, even though Tsr2-independent release of Rps26 was observed at 1 M KOAc (Fig. 2, C and D), the results here show that at moderate KOAc concentrations, the release of Rps26 is promoted by Tsr2.

To confirm that the Tsr2-mediated release of Rps26 at high salt represented a specific biological response and not just globally weakened binding of ribosomal proteins at high salt, which were then captured by their chaperones, we tested whether the Tsr2-dependent release of Rps26 was specific or also observed with other chaperone-ribosomal protein (RP) pairs. Thus, we cloned, expressed, and purified Tsr4, the chaperone for Rps2 (*32*), and Yar1, the chaperone for Rps3 (*33*), and then tested whether their addition to 40*S* subunits led to release of Rps2 and Rps3, respectively (fig. S2, C and D). Notably, while 500 mM KOAc led to Tsr2-induced release of Rps26, neither Tsr4 or Yar1 is able to release Rps2 or Rps3, respectively. Moreover, as expected, given the Tsr2 dependence for Rps26 release (Fig. 2, C and D), addition of either Tsr4 or Yar1 also does not lead to release of Rps26. Thus, the Tsr2-mediated release of Rps26 is specific and not a global effect from possibly weakened binding of ribosomal proteins at higher salt conditions.

To verify that the physical interaction between Tsr2 and Rps26 is essential for Rps26 dissociation and demonstrate a specific interaction between Tsr2 and Rps26, we wanted to confirm that Rps26 release required the characterized binding interface between Rps26 and Tsr2. Previous work has shown that the Tsr2_DWI mutant (D64W65I66A) impairs binding to Rps26 (*28*) (see also fig. S4, E and F). As expected, Tsr2_DWI was unable to dissociate Rps26 from 40*S* subunits at high salt concentrations (Fig. 2, E and F).

Similarly, the C-terminal tail of Rps26 is required for binding to Tsr2 (*28*). If Tsr2 released Rps26 from ribosomes by binding this part of the protein, we would expect that truncated Rps26^1–99^ is resistant to release from 40*S* subunit under increased level of KOAc. The release of Rps26 was more efficient from 40*S* subunits containing full-length Rps26^WT^ compared with those containing Rps26^1–99^ (Fig. 2, G and H) while still requiring Tsr2 (fig. S2E). Thus, these data demonstrate that the Tsr2-dependent release of Rps26 from 40*S* subunits requires binding of Tsr2 to Rps26 via its previously characterized binding interface.

### Tsr2 remodels mature ribosomes to generate Rps26-deficient ribosomes under stress

**Fig. 3.**
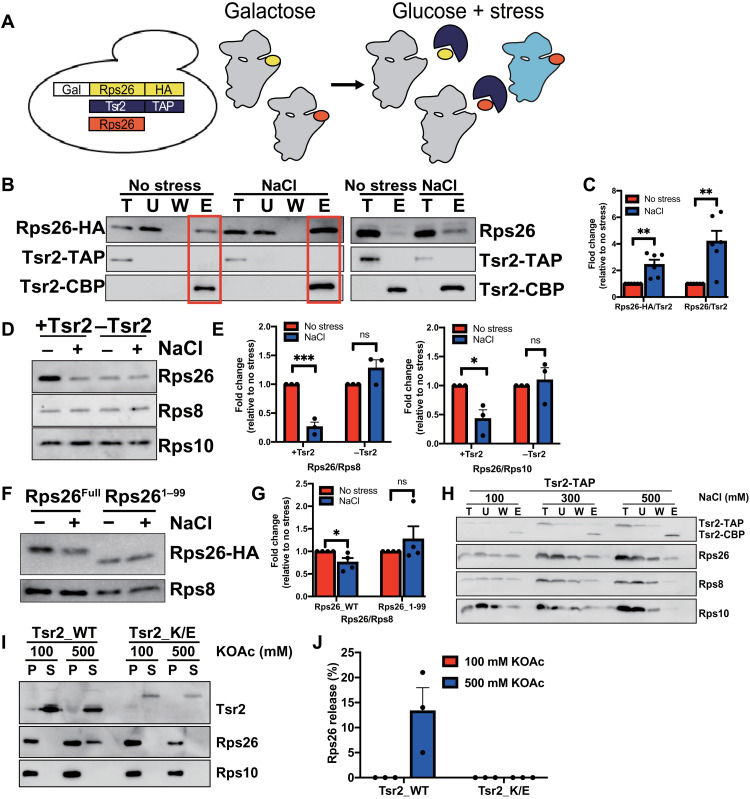
Tsr2 dissociates Rps26 from preexisting ribosomes under salt stress in vivo. (**A**) Pulse-chase experiments to follow preexisting Rps26-HA (yellow) rely on a yeast strain where Rps26-HA is produced from a galactose-inducible/glucose-repressible promoter. Tsr2 (blue) is captured with the TAP-affinity tag, and untagged Rps26 is in red. (**B**) Western blot of preexisting Rps26-HA co-isolated with TAP-tagged Tsr2 from cells treated with or without 1 M NaCl. (**C**) Quantification of six biological replicates such as in (B). (**D**) BY4741 (+Tsr2) or Gal::Tsr2 (−Tsr2) cells grown in glucose media for 18 hours with or without with 1 M NaCl. Rps8, Rps10, and Rps26 in purified ribosomes were determined by Western blot. (**E**) Quantification of three biological replicates such as in (D). (**F**) Cells containing Rps26 or Rps26^1–99^ were treated with or without 1 M NaCl. The concentrations of Rps8 and Rps26 in cell lysates were measured by Western blot. (**G**) Quantification of four biological replicates such as in (F). (**H**) Tsr2 interacts with ribosomes. IgG pulldown from cells containing TAP-tagged Tsr2, analyzed for Tsr2, Rps26, Rps8, and Rps10. U, unbound, W, wash. The same cell lysates were divided in to three columns and washed with wash buffer containing 100, 300, or 500 mM NaCl. (**I**) Rps26 release assay with the 40*S* interaction–deficient Tsr2_K/E mutant. (**J**) Quantification of Rps26 in (I) with three replicates. Error bars represent the SEM in all cases, and significance was determined using an unpaired *t* test. *P < 0.05; **P < 0.01; ***P < 0.001.

Above, we have shown that Tsr2 can release Rps26 from mature ribosomes in the presence of high salt in vitro. We next tested if Tsr2 was also involved in Rps26 release from ribosomes when yeast cells are stressed in vivo. If so, then we predict that the Rps26 released from preexisting ribosomes will be bound to Tsr2 under stress. To confirm this hypothesis, we redesigned the pulse-chase experiment by following preexisting Rps26-HA (hemagglutinin) bound to Tsr2-TAP. In this experiment, the expression of HA-tagged Rps26 is regulated by a galactose-inducible/glucose-repressible promoter (in the background of constitutive untagged Rps26). By switching the media to YP-glucose when Na^+^ is added, we ensure that only preexisting ribosomes contain Rps26-HA (Fig. 3A). If Tsr2 binds Rps26-HA released from preexisting ribosomes, then we expect that more preexisting Rps26-HA is bound to Tsr2 in stress-treated than untreated cells. Control experiments using gradient centrifugation show that Rps26-HA is bound to 40*S* subunits akin to the excess untagged Rps26 (fig. S3A).

The data in Fig. 3 (B and C) show that more of the preexisting Rps26-HA is bound to Tsr2 in stress-treated than untreated cells. The same is true for untagged Rps26, as expected, as this is a mixture of new and preexisting Rps26. Thus, these data show that preexisting Rps26 is shifted to a Tsr2-bound complex after stress, correlating with the loss of preexisting Rps26 from 40*S* subunits.

To provide evidence that Tsr2 promotes release of Rps26 and not just captures and then stabilizes Rps26 released from ribosomes in vivo, we carried out three sets of experiments: We tested (i) whether Tsr2 was required for the formation of Rps26-deficient ribosomes in vivo, (ii) whether Tsr2 bound to ribosomes, and (iii) whether 40*S* binding by Tsr2 was required for Rps26 release. To test whether Tsr2 was required for the formation of Rps26-deficient ribosomes under Na^+^ stress in vivo, we compared the amount of Rps26 in stress-treated and untreated ribosomes when Tsr2 is present or absent in those cells. Tsr2 was depleted using a galactose-inducible/glucose-repressible strain grown in glucose for ~18 hours. As previously reported (*27*), Tsr2 depletion reduced the growth rate about twofold (fig. S3B). Consistent with a role for Tsr2 in Rps26 incorporation, the resulting 40*S* subunits had somewhat less Rps26 when grown in rich medium (fig. S3C). However, when the Tsr2-depleted ribosomes were exposed to high NaCl stress, the amount of Rps26 in the subunits was no longer reduced, in contrast to what was observed in the presence of Tsr2. Instead, without Tsr2, no change or even an increase in Rps26 occupancy in ribosomes is observed (Fig. 3, D and E). These experiments demonstrate that Tsr2 is required for the reduced levels of Rps26 after Na^+^ addition in vivo. Moreover, as for the in vitro experiments, the C-terminal tail of Rps26, which binds Tsr2, is required for the change in Rps26 levels with NaCl stress, as Rps26 levels remain constant in Rps26^1–99^ cells, while they are reduced in cells containing wt Rps26 (Fig. 3, F and G). This analysis accounts for the reduced amount of Rps26 in the ribosomes from the Rps26^1–99^ cells (fig. S3D). Thus, the interaction between Rps26 and Tsr2 is required for Rps26 release in vivo.

To test whether Tsr2 interacted with 40*S* ribosomes, as expected, if it releases Rps26 from these subunits, we repeated the Tsr2-TAP pulldown and washed the IgG beads with different salt concentrations after Tsr2-TAP had been bound (Fig. 3H). The conditions under which the cells were grown, lysed, or bound to the TAP-resin were unchanged. These experiments show that Tsr2-TAP copurifies 40*S* ribosomes and that these are loosely bound and washed off when the salt in the wash buffer is raised to 500 mM NaCl. This is not simply a contamination of the IgG resin, due to the abundance of ribosomes, as the transcription factor Pos9 does not copurify ribosomes (fig. S3E). Rps26 remains bound to Tsr2, even under these higher Na^+^ conditions. Similarly, gradient centrifugation experiments also demonstrate that a fraction of Tsr2 interacts with 40*S* subunits alone, or when in 80*S* fractions or even polysomes (fig. S3A). Thus, Tsr2 binds 40*S* subunits.

Next, we tested whether the binding of Tsr2 to 40*S* subunits was required for release of Rps26, as predicted from a model where Tsr2 actively releases Rps26, as opposed to simply capturing released Rps26. For that purpose, we generated a Tsr2 mutant, where a positively charged patch in the protein was mutated to generate Tsr2_K/E (K18E;K75E;K78E;K42E;K127E;K130E;R140E;K142E;K143E;K145E;R146E). This mutant displays a slow growth phenotype (fig. S4A). Gradient centrifugation of yeast lysates demonstrates that Tsr2_K/E was unable to bind 40*S* subunits (fig. S4D), while both wt Tsr2 and Tsr2_DWI cosediment with the subunits (fig. S4, B and C). In contrast, the Tsr2_K/E mutant, but not the Tsr2_DWI mutant, retained the ability to bind Rps26 (fig. S4, E and F).

Using this mutant, we next tested whether it was able to release Rps26 from 40*S* subunits. This mutant was unable to release Rps26 from purified 40*S* subunits (Fig. 3, I and J), as we have shown for the Tsr2_DWI mutant.

Thus, together, these data demonstrate that Tsr2 can release Rps26 in vitro and that its ability to bind both Rps26 and the 40*S* subunit is required for Rps26 release, demonstrating that Tsr2 does not just simply capture released Rps26 but providing evidence for an active role in Rps26 release.

### Rps26-deficient ribosomes are repaired to mature ribosomes after stress

Above, we have shown that during high Na^+^ stress, Rps26 is released from preexisting ribosomes by Tsr2, to which it remains bound. In addition, previous work suggested that Tsr2 is involved in the incorporation of Rps26 into ribosomes. We therefore wondered whether the Tsr2-dependent release of Rps26 was reversible, such that Rps26 could be reincorporated from the Tsr2-bound complex into ribosomes once stress was removed. This would spare ribosomes whose assembly requires extensive resources from destruction while also helping cells to quickly return to the normal translational program.

**Fig. 4.**
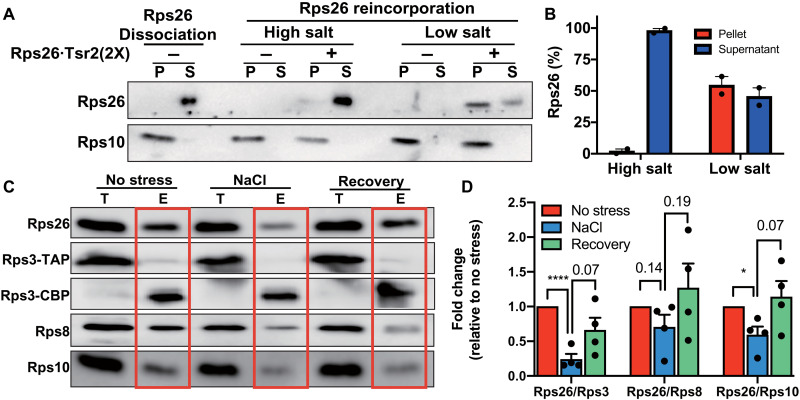
Repair of Rps26-deficient ribosomes after stress. (**A**) Rps26 cosedimentation with ribosomes after incubation of Rps26-deficient ribosomes with recombinant Rps26•Tsr2 in vitro. Western blot analysis of ultracentrifugation pelleting experiments. Rps26-deficient ribosomes (Rps26 dissociation) were generated by Tsr2 addition in 750 mM KOAc and separated from free Rps26•Tsr2 by ultracentrifugation. Rps26-deficient ribosomes were incubated with purified recombinant Rps26•Tsr2 in low (100 mM KOAc) or high (750 mM KOAc) salt buffers, and Rps26 binding was assessed after pelleting of the ribosomes. S, supernatant; P, pellet. (**B**) Data from two independent experiments were quantified. (**C**) Rps26 is reinserted into ribosomes in vivo. To monitor the repair of Rps26-deficient ribosomes by reinsertion of Rps26, the same pulse-chase experiment as in Fig. 1 was performed, except NaCl stress was relieved by dilution into fresh media. (**D**) Quantification of data in (C). Data are averages from four biological replicates. Error bars represent the SEM, and significance was determined using an unpaired *t* test. *P < 0.05; ****P < 0.0001.

To test whether Rps26-deficient ribosomes can reincorporate Rps26 in vitro, we took advantage of our release assay. We first generated 40*S* subunits lacking Rps26 by incubating 40*S* subunits with Tsr2 in high salt and separating them from released Rps26•Tsr2 (Fig. 4A; Rps26 dissociation). We then added recombinant Rps26•Tsr2 to the Rps26-deficient ribosomes in low (100 mM) or high (750 mM) K^+^. Rps26 was reincorporated only when the K^+^ concentration was lowered, showing that Rps26 can assemble into Rps26-deficient ribosomes from the Rps26•Tsr2 complex (Fig. 4, A and B). Moreover, we note that regeneration is complete, as half of Rps26 was observed in the pellet after addition of a twofold excess in the Rps26•Tsr2 complex.

To test whether Rps26-deficient ribosomes can be repaired by reincorporation of Rps26 from Rps26•Tsr2 in vivo, we extended the pulse-chase experiment in Fig. 1. As before, we first generated Rps26-deficient 40*S* subunits by addition of high NaCl to yeast cells (Fig. 4, C and D; NaCl), before removing the stress for 1 hour. After this recovery, Rps26 levels in preexisting ribosomes also recovered (Fig. 4, C and D; recovery), showing that Rps26 can be reincorporated into preexisting ribosomes from which it had been previously released.

Together, the results here suggest that Tsr2 releases Rps26 from ribosomes when exposed to high NaCl to form a Rps26•Tsr2 complex. Moreover, the data also indicate that once the NaCl is removed, these ribosomes can be repaired by reincorporation of Rps26 from the Rps26•Tsr2 complex.

### Ribosomes directly sense increased Na^+^/pH

Above, we have shown that addition of 1 M NaCl leads to the release of Rps26 from ribosomes to Tsr2 in vivo, which can be reversed when the stress is removed. We next wondered whether the function of Tsr2 in either releasing or incorporating Rps26 into 40*S* subunits was simply a function of the salt concentration, or pH, or perhaps required additional signaling cascades, resulting in posttranslational modifications such as phosphorylation or ubiquitination. To search for evidence of large posttranslational modifications, such as ubiquitination, in vivo we used Western blotting after salt exposure. However, these data do not provide any evidence for ubiquitination of either Rps26 or Tsr2 when yeast cells were treated with NaCl stress (fig. S5A). Moreover, no increase in phosphorylation of Rps26 or Tsr2 was observed when phosphorylation of the total proteome was analyzed after NaCl treatment (*34*), and we were unable to detect phosphorylation of Rps26 or Tsr2 using phosphoserine antibodies (fig. S5, B and C). Last, mass spectrometry also did not reveal any posttranslational modifications in Rps26. While these (or any other) data cannot rule out a distinct posttranslational modification, they led us to consider whether the ribosome could directly sense an increase in the salt concentration or pH, becoming susceptible to Tsr2-mediated extraction even at physiological Na^+^ concentrations. This model would be consistent with the previous observation that the Rps26•Tsr2 complex was strengthened at high salt concentrations (*28*), while the Rps26•40*S* interaction is weakened (Fig. 2C).

While the data above show that Tsr2-mediated Rps26 release required about 500 mM KOAc, the concentrations of Na^+^ and K^+^, the two most abundant cations in yeast cells, are ~20 and ~200 mM, respectively (*35*). Addition of 1 M NaCl to the media leads to an increase in the intracellular Na^+^ concentration to ~150 mM, while the total concentration of Na^+^ and K^+^ is maintained at ~300 mM by decreasing the intracellular level of K^+^ to ~150 mM (*35*, *36*). We therefore wondered whether Na^+^ and K^+^ had different concentration dependences for Rps26 release, reflecting different affinities, as expected from an organized binding site that has a regulatory function.

**Fig. 5.**
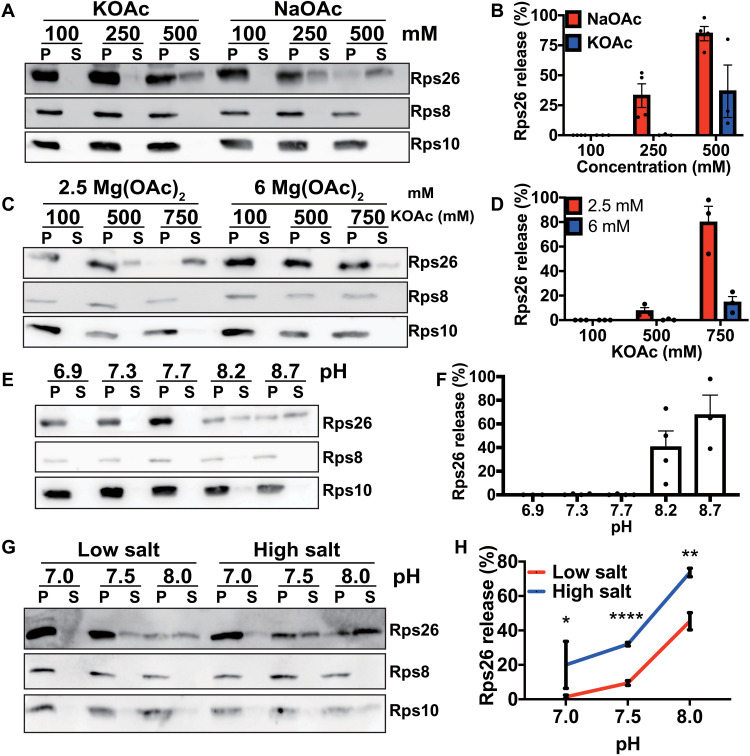
Ribosomes directly sense ion and protein concentrations in vitro. (**A**) Western blot analysis of pelleted (P) ribosomes and released proteins in the supernatant (S). Mature 40*S* subunits purified from yeast were incubated with recombinant Tsr2 at different concentrations of NaOAc or KOAc. (**B**) Quantification of Rps26 in (A) with three to four independent replicates for each condition. Error bars represent the SEM. (**C**) Effect of Mg^+^ in the release of Rps26. Mature 40*S* subunits were incubated with recombinant Tsr2 in either 2.5 or 6 mM MgOAc at different KOAc concentrations. (**D**) Quantification of Rps26 in (C) with three independent replicates for each condition. Error bars represent the SEM. (**E**) Release assay in 20 mM bis-tris propane at various pH values, 2.5 mM MgOAc and 100 mM KOAc. (**F**) Quantification of Rps26 in (E) with three to four independent replicates for each condition. Error bars represent the SEM. (**G**) Release of Rps26 under physiological conditions. Mature 40*S* subunits were incubated with recombinant Tsr2 in either low physiological salt conditions (20 mM NaCl, 200 mM KCl, and 2.5 mM MgOAc) or high physiological salt concentrations (150 mM NaCl, 150 mM KCl, and 2.5 mM MgOAc) with different pH values adjusted by bis-tris propane. (**H**) Quantification of results in (G) with three to nine independent replicates for each condition. Error bars represent the SEM. Significance was determined using an unpaired *t* test. *P < 0.05; **P < 0.01; ****P < 0.0001.

To test this hypothesis, we compared Na^+^ and K^+^ effects on Rps26 release. The data show that Na^+^ concentrations that lead to Rps26 dissociation are much lower than K^+^ concentrations (Fig. 5, A and B, and fig. S6A) and within the range of physiological intracellular concentrations under high salt stress.

To further dissect how K^+^ or Na^+^ affected Rps26 binding, we next tested the model that K^+^ or Na^+^ evicts an Mg^2+^ ion stabilizing Rps26 binding. This hypothesis was motivated by the observation that there are several ions bound to the Rps26-40*S* interface (fig. S7A) (*37*). Thus, we repeated the release assay at higher Mg^2+^ concentrations. Increasing the Mg^2+^ concentration from 2.5 to 6 mM largely blocks the Na^+^- and Tsr2-dependent release of Rps26 (Fig. 5, C and D, and fig. S6B), supporting the model that increased intracellular salt concentrations lead to Rps26 release from 40*S* subunits via competition with an Mg^2+^ ion critical for Rps26 binding.

In addition to NaCl stress, alkaline pH stress also promoted the formation of Rps26-deficient ribosomes in yeast cells (*12*). Therefore, we further tested whether pH changes could also trigger Tsr2-dependent Rps26 release in vitro. Increasing the pH of the growth media to pH 8.2 will increase the intracellular pH to ≥7.5 [(*38*); as the authors note, the measured pH 7.5 is likely an underestimate]. We therefore varied the pH in release experiments between pH 6.9 and pH 8.7. Increasing the pH leads to Tsr2-dependent Rps26 dissociation from mature ribosomes (Fig. 5, E and F, and fig. S6C). These data strongly suggest that changes in pH, similar to changes in Na^+^ concentration, are also directly recognized by the ribosome, leading to Rps26 dissociation.

Last, we asked whether changes in pH and salt were acting via the same residues or different residues, which would make these changes in the cellular environment additive. To answer this question, we tested whether Rps26 release was affected by pH changes on top of ion changes, or whether, at high pH, salt would no longer matter. As shown in Fig. 5 (G and H), changes in pH and salt are additive, suggesting that they act via distinct residues at the 40*S*•Rps26 interface. Moreover, these observations also indicate that the response to these physiological changes is synergistic, such that lower salt is required when the pH is also elevated. Last, the data show that under physiological low salt conditions, the elevated pH 7.5 observed in pH stress cells can effect Tsr2-dependent Rps26 release, while at pH 7.0, the physiological high salt conditions lead to Rps26 release (Fig. 5, G and H).

## DISCUSSION

### Tsr2-dependent remodeling and repair of ribosomes by reversible release of Rps26

**Fig. 6.**
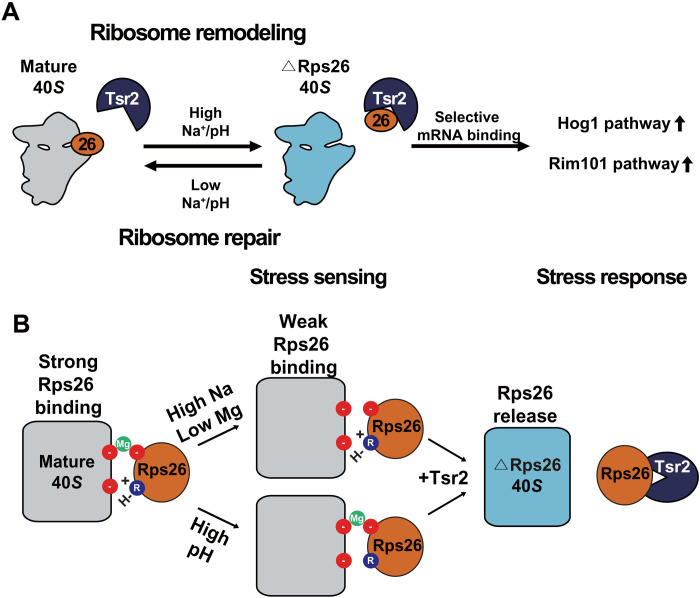
Model for the Tsr2-mediated remodeling and repair of Rps26-deficient ribosomes. (**A**) High intracellular Na^+^ concentrations or pH leads to ribosome remodeling by Tsr2-dependent dissociation of Rps26 from 40*S* ribosomes, producing Rps26-deficient ribosomes that display altered mRNA preference and enable translation of mRNAs from the Hog1 and Rim101 salt and pH response pathways (*12*). Removal of the stress allows for ribosome repair by allowing reincorporation of Rps26 from the Rps26·Tsr2 complex. (**B**) Rps26 binding to 40*S* ribosomes is Mg^2+^ and H^+^ dependent and involves two distinct sites.

We have previously shown that yeast cells exposed to high Na^+^ or high pH stress accumulate ribosomes lacking Rps26 (*12*). Here, we show that sorbitol stress also leads to Rps26 release. The resulting Rps26-depleted ribosomes enable translation of mRNAs with an otherwise unfavorable G at position −4 in the Kozak sequence, thereby promoting a distinct translational program that supports the response to these stresses (*12*). Here, we address how these Rps26-deficient ribosomes are formed (Fig. 6A).

Our data show that high salt or pH affects the 40*S* subunit directly, weakening the binding of Rps26 such that it can be released by Tsr2. To effect this function, Tsr2 must bind the 40*S* subunits and Rps26, and disrupting either of these interactions abrogates Tsr2’s ability to release Rps26. Thus, Rps26-deficient ribosomes are formed by binding of Tsr2 to Rps26 incorporated into preexisting 40*S* ribosomes to release Rps26, and not simply by capture of released Rps26 by Tsr2. Nonetheless, once released, Rps26 remains bound to Tsr2, which thus stabilizes it outside of the ribosome. This Na^+^- or pH-dependent release of a ribosomal protein by its chaperone is specific to the Tsr2/Rps26 chaperone/ribosomal protein pair and not observed for the Tsr4/Rps2 and Yar1/Rps3 pairs.

Moreover, we also demonstrate that Rps26 can be incorporated from the Rps26•Tsr2 complex into Rps26-deficient ribosomes in vivo and in vitro, thereby providing direct evidence for a role of this chaperone in the incorporation of Rps26. Such a role was previously suggested on the basis of the observation that Tsr2 stabilizes Rps26 outside of the ribosome (*27*) and that the growth defect from Tsr2 deletion can be suppressed by Rps26 overexpression (*28*).

Together, these results suggest that under stress, Tsr2 remodels 40*S* ribosomal subunits to release Rps26, which is stored in the Rps26•Tsr2 complex, from which it can be reincorporated to repair the subunits after the stress subsides (Fig. 6A). To our knowledge, this is the first instance of active remodeling and repair of ribosomes, prompting us to speculate that ribosome remodeling could be a more common way to rapidly generate different ribosome populations and that repair of ribosomes damaged by age (e.g., in oocytes) or by oxidative stress could be observable in other instances.

### Ribosomes as sensors for fluctuations in intracellular salt, Mg^2+^, and pH

The results in Fig. 5 show that the mature 40*S* subunit itself can detect variations in Mg^2+^, Na^+^, or H^+^ concentrations, triggering the Tsr2-enabled dissociation of Rps26. The Rps26-deficient ribosomes then function by enabling the translation of mRNAs with an otherwise disfavored −4G mutation in the Kozak sequence, which changes protein homeostasis (*12*). Thus, the ribosome is both a sensor for physiological changes in intracellular salt, Mg, and pH, as well as a mediator for responding to these changes (Fig. 6A).

How do ribosomes sense differences in salt and pH? Release assays at different Mg^2+^ concentrations strongly suggest that the release of Rps26 by Na^+^ occurs via competition with an Mg^2+^ ion that stabilizes Rps26 binding, either directly by bridging the RNA and protein or by stabilizing the RNA structure (Fig. 6B). Crystal structures identify multiple Mg^2+^ ions near the Rps26-binding site (*37*), including one bound to Asp33, an essential residue conserved in human Rps26 [fig. S7, A and B; at 3-Å resolution, it is impossible to distinguish Mg^2+^ from K^+^ ions; however, the crystal well contains fairly high concentrations of Mg^2+^ (3.3 to 10 mM) and low concentrations of K^+^ (95 mM)]. This Mg^2+^ also contacts the rRNA backbone and could thus be a candidate ligand for mediating the salt-dependent effect. The D33N mutation leads to DBA (*39*) but does not affect the interaction with Tsr2 (*27*). We thus hypothesized that the D33N mutation weakens Rps26 binding via loss of the Mg^2+^-binding site. Purification of 40*S* subunits from cells containing Rps26_D33N shows that these have lost nearly all Rps26 (fig. S7C), demonstrating the importance of the metal ion at that position for Rps26 binding. Because these ribosomes entirely lack Rps26, we could not directly test whether Rps26 release displayed a different response to high salt. Nonetheless, we note that there are two additional ions within less than 7 Å to the D33-bound ion (fig. S7A). It is conceivable that the differential occupation of these sites by Na^+^, K^+^, and Mg^2+^ is responsible for the observed salt effects on Rps26 release.

If ribosomes are direct sensors for cellular pH, Na^+^, and Mg^2+^, how then do we explain the sorbitol-dependent release of Rps26? Mathematical modeling of cellular responses to different ionic changes predicts that sorbitol reduces the cellular volume by water extrusion, leading to an increase in intracellular Na^+^ concentration and effectively producing intracellular NaCl stress upon exposure to sorbitol (*40*), a prediction confirmed in microscopy studies (*41*, *42*). In contrast, the same simulations indicate that high pH stress does not change the intracellular NaCl concentrations, thus explaining why the ribosomes must have a separate pH sensor. Whether the effect on ribosomes arising from the exposure to high salt and sorbitol is identical remains unclear; however, quantitative differences in the response would be consistent with both our data and previous observations (*43*, *44*). It should also be noted that we have not explored whether the fate of the Tsr2-bound Rps26 is the same under conditions of high NaCl, sorbitol, and pH stress.

Two lines of evidence also suggest that salt and pH act independently of each other, indicating that the pH-sensitive site is distinct from the salt-dependent site. First, the pK_a_ value for the ionizable group(s) is above 8, implicating side chains such as lysines and histidines. While histidines can provide ligands to soft metals such as Zn in Zn-finger motifs, neither histidine nor lysine is a typical ligand for Mg ions, thus suggesting that the pH effects are mediated by different side chains than the salt effects, which possibly arise from D33 as discussed above. Moreover, our data indicate that the effects from pH and salt are additive, although the strength of this conclusion is limited because we cannot fully deprotonate the residue that is responsible for the pH dependence.

### Remodeling preexisting ribosomes might be advantageous under stress

Transcriptional responses to remodel the proteome under stress are well characterized and occur rapidly (*30*). Thus, the advantages of changing the ribosome population are not immediately clear but could include the ability to produce different branches of a stress response by overlaying it onto changes in the mRNA population (*13*, *19*, *45*) and the potential to preferentially boost production of the stress proteome while producing moderate, instead of very high, amounts of the corresponding mRNAs. Nonetheless, the potential disadvantage, the need to turn over an entire ribosome population at great energetic cost, is clear. This is especially puzzling because energy supplies are often limited under cellular stress and because stress generally stops ribosome transcription and assembly (*1*, *30*). Moreover, the turnover of ribosomes is also expected to be much slower than the turnover of mRNAs, thereby rendering such a response relatively slow (*26*, *46*). Here, we show how these disadvantages can be neutralized by remodeling preexisting ribosomes to rapidly produce distinct ribosome populations without any additional energy input. Notably, ribosome remodeling also solves an additional potential problem, the existence of quality control pathways in place to ensure that ribosomes are correctly assembled (*2*–*6*). Presumably, these mechanisms limit the accumulation of not only ribosomes lacking head components (*6*) but also those lacking Rps26. By generating Rps26-deficient ribosomes from fully and correctly assembled subunits, these quality control mechanisms do not need to be circumvented, which could open the cells to the uncontrolled production of Rps26-deficient ribosomes, which are associated with DBA (*47*).

## METHODS

### Strains and plasmids

*Saccharomyces cerevisiae* strains used in this study were either purchased from the GE Dharmacon Yeast Knockout Collection or constructed using standard methods (*48*) and are listed in table S1. Plasmids are listed in table S2.

### Protein purification

Rps26, Tsr2, Tsr2_DWI, Tsr2_K/E, Tsr4, and Yar1 were expressed in *Escherichia coli* Rosetta2 (DE3) cells (Novagen) as tobacco etch virus (TEV)–cleavable His6-MBP (polyhistidine–maltose-binding protein) fusion proteins. Cells were grown at 37°C in LB (Luria-Bertani) medium supplemented with antibiotics. At OD_600_ (optical density at 600 nm) of 0.4, protein expression was induced with 1 mM isopropyl-β-d-thiogalactopyranoside (IPTG) for 16 hours at 18°C. Proteins were purified using Ni-NTA resin (Qiagen) according to the manufacturer’s instructions. Eluted proteins were pooled and dialyzed overnight at 4°C into 50 mM tris (pH 7.4), 100 mM NaCl, and 1 mM dithiothreitol (DTT) with TEV protease. Tsr2, Tsr4, and Yar1 were further purified by MonoQ and Superdex 75 (GE) chromatography. For purification of the **Rps26·Tsr2 complex, the His-MBP tag was removed by a second round of purification with Ni-NTA resin, and the Rps26•Tsr2 complex was further purified by Superdex 75 with complex buffer [50 mM tris (pH 7.4) and 500 mM NaCl]. Concentrated proteins were stored at −80°C.

### Mass spectrometry

To obtain the exact mass of purified recombinant Tsr2_WT and Tsr2_K/E, buffer exchange to dH_2_O was performed with purified recombinant proteins using Zeba spin desalting columns (Thermo Fisher Scientific). Next acetonitrile and formic acid were added to final concentrations of 50 and 0.1%, and the protein was analyzed by electrospray ionization mass spectrometry. The spectra were analyzed by Q exactive (Thermo Fisher Scientific).

### Lysis of yeast cells

All yeast cells were harvested, washed, and then resuspended in cell pellet (1 ml/g) of the appropriate lysis buffer. The suspension was frozen by dripping into liquid nitrogen to produce pearls. These were then grounded with mortar and pestle under liquid N_2_. The resulting powder was stored at −80°C until use. At that time, another cell pellet (1 ml/g) of the appropriate lysis buffer was added together with about 0.5 g of beads, and the mixture was thawed while rotating. Cell debris and beads were removed by centrifugation for 10 min at 3000*g*, and the supernatant was clarified by centrifugation for 10 min at 20,000*g*.

### Isolation of Rps3-TAP–tagged premade ribosomes

One liter of cells was grown to mid-log phase in YPD to produce ribosomes with Rps3-TAP, before shifting them to YPGal in the presence of dox (0.2 μg/ml), to shut off Rps3-TAP and induce untagged Rps3. At the same time, half of the cells were also treated with 1 M NaCl, and all cells were grown for four more hours. For sorbitol stress, cells were treated with 1 M sorbitol and grown for 30 additional minutes. To test the repair of Rps26-deficient ribosomes, cells were further grown in YPGal for 1 hour after stress in the presence of dox.

For TAP purification of the lysate, ~200 μl of prewashed IgG sepharose bead slurry (GE) was added to each lysate and incubated for ~2 hours at 4°C. After binding, each sample was washed with IgG-binding buffer [50 mM tris (pH 7.5), 100 mM NaCl, 10 mM MgCl_2_, 0.075% NP-40, 1 mM benzamidine, and 1 mM phenylmethylsulfonyl fluoride (PMSF)] three times before elution. Elution step was performed with ~2 hours of incubation in 16°C with IgG-binding buffer supplemented with TEV protease (1:100; Invitrogen), 0.5 mM EDTA, and 1 mM DTT. Samples were further analyzed using Western blot.

### Isolation of Rps26 bound to Tsr2 after stress

Tsr2-TAP cells with pKK30528 (Gal:Rps26-HA) were grown to mid-log phase in YPGal media before expression of Rps26-HA was repressed by growth in YPD for 2 hours. Cells were then split into two pools and grown for 4 hours in YPGal with or without 1 M NaCl. After collecting the cells, TAP purification was performed as above, except that 500 mM NaCl was added to the wash buffer to remove ribosomes bound to Tsr2.

### In vitro Rps26 release assay

Pelleting release assays were performed as previously described (*49*). Briefly, 4 μM purified recombinant chaperone (Tsr2, Tsr2_DWI, Tsr2_E_mutant, Tsr4, or Yar1) was mixed with 40 nM 40*S* subunits purified as described below, incubated for 15 min at room temperature and 10 min on ice in binding buffer [20 mM Hepes (pH 7.3), 2.5 mM MgOAc, heparin (0.1 mg/ml), 2 mM DTT, and 0.5 μl of RNasin (NEB)] containing different concentrations of salt or magnesium. Samples were layered onto a 400-μl sucrose cushion [ribosome-binding buffer and 20% sucrose (w/v)] and spun for 2.5 hours at 400,000*g* at 4°C in a TLA 100.1 rotor (Beckman). Supernatants were precipitated using trichloroacetic acid and resuspended in the same volume as pellets. Total resuspended sample was loaded on SDS–polyacrylamide gel electrophoresis (SDS-PAGE) followed by Western blotting. For pH-dependent release assays, bis-tris propane was used to adjust the pH to the indicated value.

### Quantitative reverse transcription polymerase chain reaction

Transcriptional repression of dox-repressible Rps3-TAP was assessed by inoculating YPGal media [with or without dox (0.2 μg/ml)] with a preculture grown in YPD to mid-log phase. Ten milliliters of cells was harvested at different time points. Harvest cells were resuspended in 400 μl of TES [10 mM tris HCl (pH 7.5), 10 mM EDTA, and 0.5% (w/v) SDS] buffer, and total RNA was isolated by hot-phenol extraction. After RNA extraction, each sample was precipitated and resuspended in 50 μl of deoxyribonuclease (DNase) master mix [5 μl of 10× RQ1 DNase Buffer (Promega), 1 μl of RQ1 DNase enzyme (Promega), and 44 μl of dH_2_O] to further remove DNA. Then, each sample was resuspended in 100 μl of H_2_O, and total RNA concentration was measured. One microgram of total RNA was used for reverse transcription using Protoscript II (New England Biolabs) per the manufacturer’s instructions, generating complementary DNA. Quantitative polymerase chain reaction (qPCR) was performed with Excella 2× SYBR master mix per the manufacturer’s instructions using primers listed in table S3.

### Gradient centrifugation

pKK30528 (pRS426-Gal::Rps26A-HA) containing YKK856 (Tsr2-TAP) cells were grown in galactose media to coexpress Rps26-HA in addition to endogenous Rps26. For experiments with YKK1109 (Gal::Tsr2) containing Tsr2 variant plasmids, cells were grown in YPD media. At mid-log phase, cycloheximide (0.1 mg/ml) was directly added to culture, and cells were harvested by centrifugation. Cell pellet was further washed and lysed in gradient buffer [20 mM Hepes (pH 7.4), 5 mM MgCl_2_, 100 mM KCl, and 2 mM DTT] supplemented with cycloheximide (0.1 mg/ml), 1 mM benzamidine, 1 mM PMSF, and complete protease inhibitor cocktail (Roche). Cleared lysate was applied to 10 to 50% sucrose gradients, centrifuged in an SW41Ti rotor for 2 hours at 40,000 rpm, and then fractionated as in figs. S3A and S4 (B to D). Western blot was performed to probe proteins as indicated.

### Purification of ribosomes

Three milliliters of each clarified lysate in ribosome buffer [20 mM Hepes/KOH (pH 7.4), 100 mM KOAc, and 2.5 mM Mg(OAc)_2_] supplemented with heparin (1 mg/ml), 1 mM benzamidine, 1 mM PMSF, and complete protease inhibitor cocktail (Roche) was layered over 500 μl of sucrose cushion (ribosome buffer, 500 mM KCl, 1 M sucrose, and 2 mM DTT) and spun in a Beckman TLA 110 rotor at 70,000 rpm for 65 min. The resulting pellet was resuspended in high salt buffer [ribosome buffer, 500 mM KCl, heparin (1 mg/ml), and 2 mM DTT], layered over 500 μl of sucrose cushion, and spun in a Beckman TLA 110 rotor at 100,000 rpm for 70 min. The flowing pellet was resuspended in ribosome storage buffer (ribosome buffer, 250 mM sucrose, and 2 mM DTT) and analyzed on SDS-PAGE followed by Western blotting.

For further subunit separation, the ribosome pellet after the second centrifugation was resuspended in subunit separation buffer [50 mM Hepes/KOH (pH 7.4), 500 mM KCl, 2 mM MgCl_2_, and 2 mM DTT], and 1 mM puromycin (Sigma-Aldrich) was added. Subunits were isolated by loading onto 5 to 20% sucrose gradients [50 mM Hepes/KOH (pH 7.4), 500 mM KCl, 5 mM MgCl_2_, 2 mM DTT, and 0.1 mM EDTA] and centrifuged at 19,600 rpm for 16 hours. 40*S* subunits were collected, and buffer was exchanged into ribosome storage buffer during concentration.

### Western analyses and antibodies

Western blots were scanned using the ChemiDoc MP Imaging System from Bio-Rad after applying luminescence substrates (Invitrogen) and quantified using its built-in image laboratory software (v 6.0.1). Intensity of each band was analyzed after local background subtraction. To detect TAP-tagged (Rps3 or Tsr2) or HA-tagged proteins (Rps26 variants), anti-TEV cleavage site from Invitrogen (PA1-119) or anti-HA antibody from Abcam (ab18181) or Sigma-Aldrich (ab1603) was used, respectively. For Rps10 and Rps26 detection, antibodies were raised by New England Peptide. Polyclonal antibodies were gifts from V. Panse (Tsr2/Rps26), A. Link (Asc1), M. Seedorf (Rps3), and J. Warner (Rps2).

## Notes

View/request a protocol for this paper from *Bio-protocol*.
