# Supplementary material for: The chaperone Tsr2 regulates Rps26 release and reincorporation from mature ribosomes to enable a reversible, ribosome-mediated response to stress
Source: Sci Adv. 2022 Feb 25;8(8):eabl4386. doi: 10.1126/sciadv.abl4386 (PMC8880767; doi:10.1126/sciadv.abl4386)
Supplement: Supplementary file 1 — Figs. S1 to S7 Tables S1 to S3 [file sciadv.abl4386_sm.pdf]

Science Advances
AAAS

Supplementary Materials for

The chaperone Tsr2 regulates Rps26 release and reincorporation from mature ribosomes to enable a reversible, ribosome-mediated response to stress

Yoon-Mo Yang and Katrin Karbstein*

*Corresponding author. Email: kkarbst@scripps.edu

Published 25 February 2022, *Sci. Adv.* **8**, eabl4386 (2022)
DOI: 10.1126/sciadv.abl4386

This PDF file includes:

Figs. S1 to S7
Tables S1 to S3

FigureS1

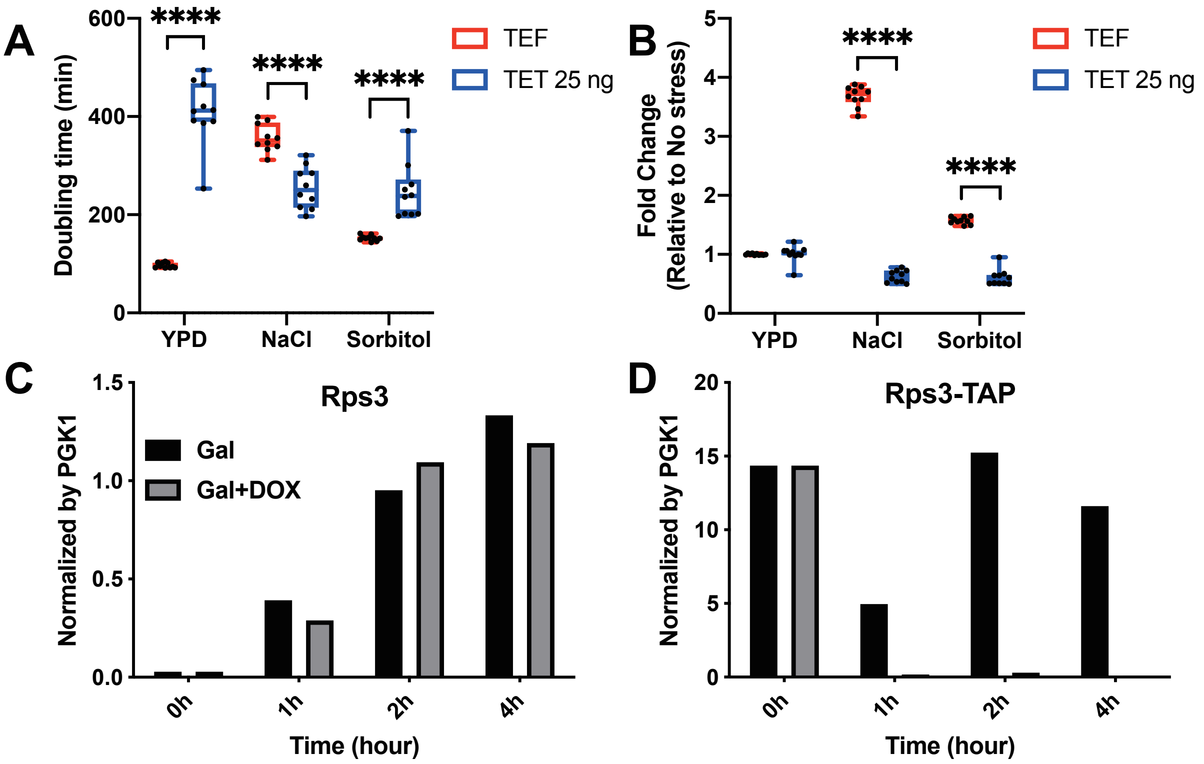

Figure S1: Rps26-deficient ribosomes are resistant to sorbitol stress. (A) Changes in doubling time upon addition 1 M NaCl or 1 M sorbitol, in YKK491 cells (Gal:Rps26) with Rps26 produced from plasmids driven by either the TEF promoter or the dox-repressible TET promoter, in the presence of 25 ng/ml dox. Data are the average of five biological replicates and two technical replicates. ****$p < 0.0001$ by unpaired t-test. (B) Values in (A) normalized to no-stress conditions (fold change = 1). ****$p < 0.0001$ by unpaired t-test. (C) Rps3 and (D) Rps3-TAP mRNA induction and repression upon galactose and doxycycline (dox) addition. Rps3 and Rps3-TAP mRNA levels were normalized to PGK1 mRNA levels as measured by qRT-PCR. Cells grown in glucose media were switched to galactose media in mid-log phase with or without doxycycline. qPCR primers were designed to differentiate between Rps3 and Rps3-Tap (**Table S3**).

FigureS2

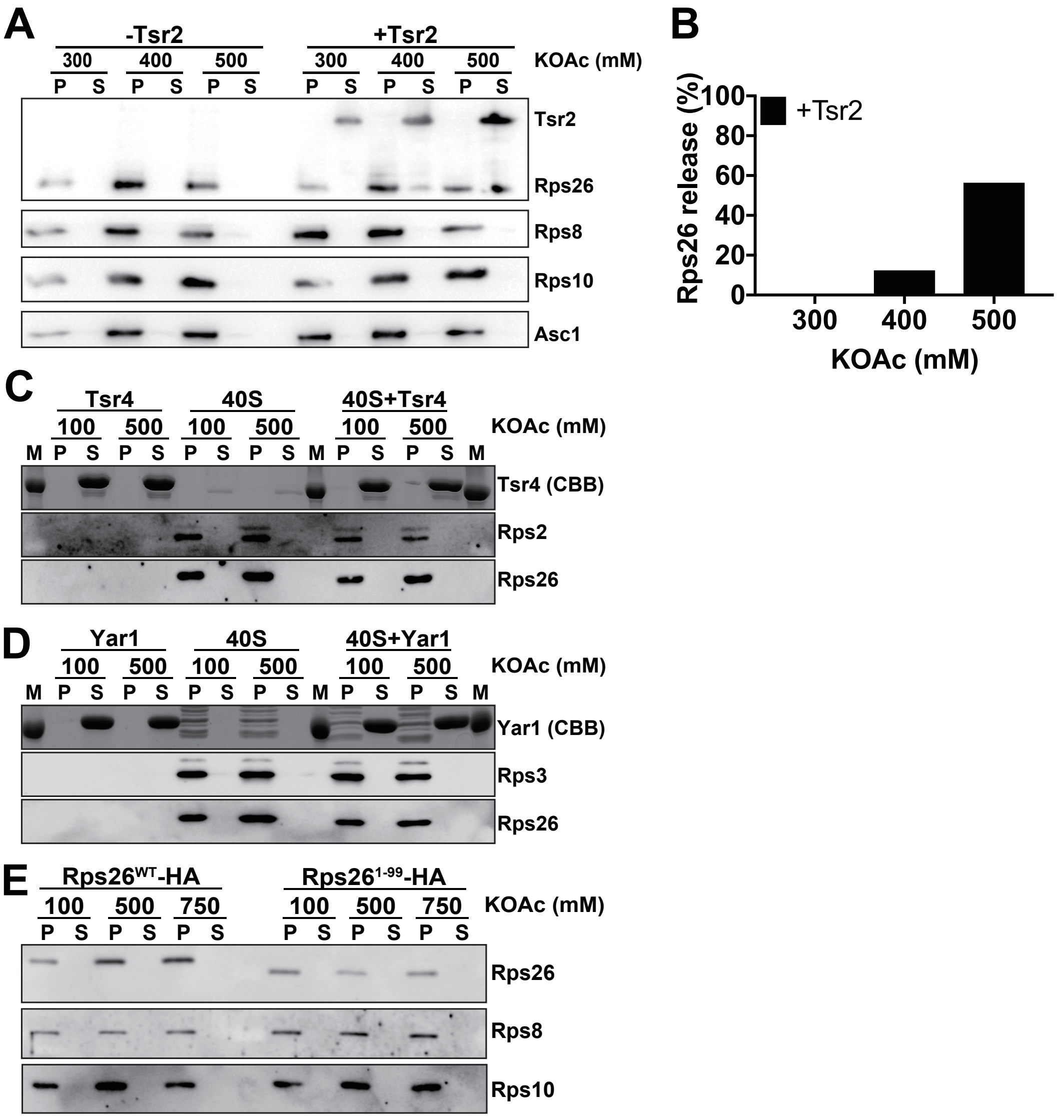

Figure S2: Release of Rps26 by Tsr2 under increased salt. (A) Release of Rps26, Rps8, Rps10 and Asc1 was measured in presence and absence of Tsr2 in different potassium concentrations by Western Blot. (B) Quantification of Rps26 in panel (A). (C) Tsr4 addition to purified 40S ribosomes does not lead to release of Rps2, or Rps26. (D) Yar1 addition to purified 40S ribosomes does not lead to release of Rps3, or Rps26. (E) Release of truncated Rps26$^{1\text{-}99}$ requires Tsr2. Control experiment for Figure 2G, showing that high salt alone does not lead to release of truncated Rps26$^{1\text{-}99}$.

FigureS3

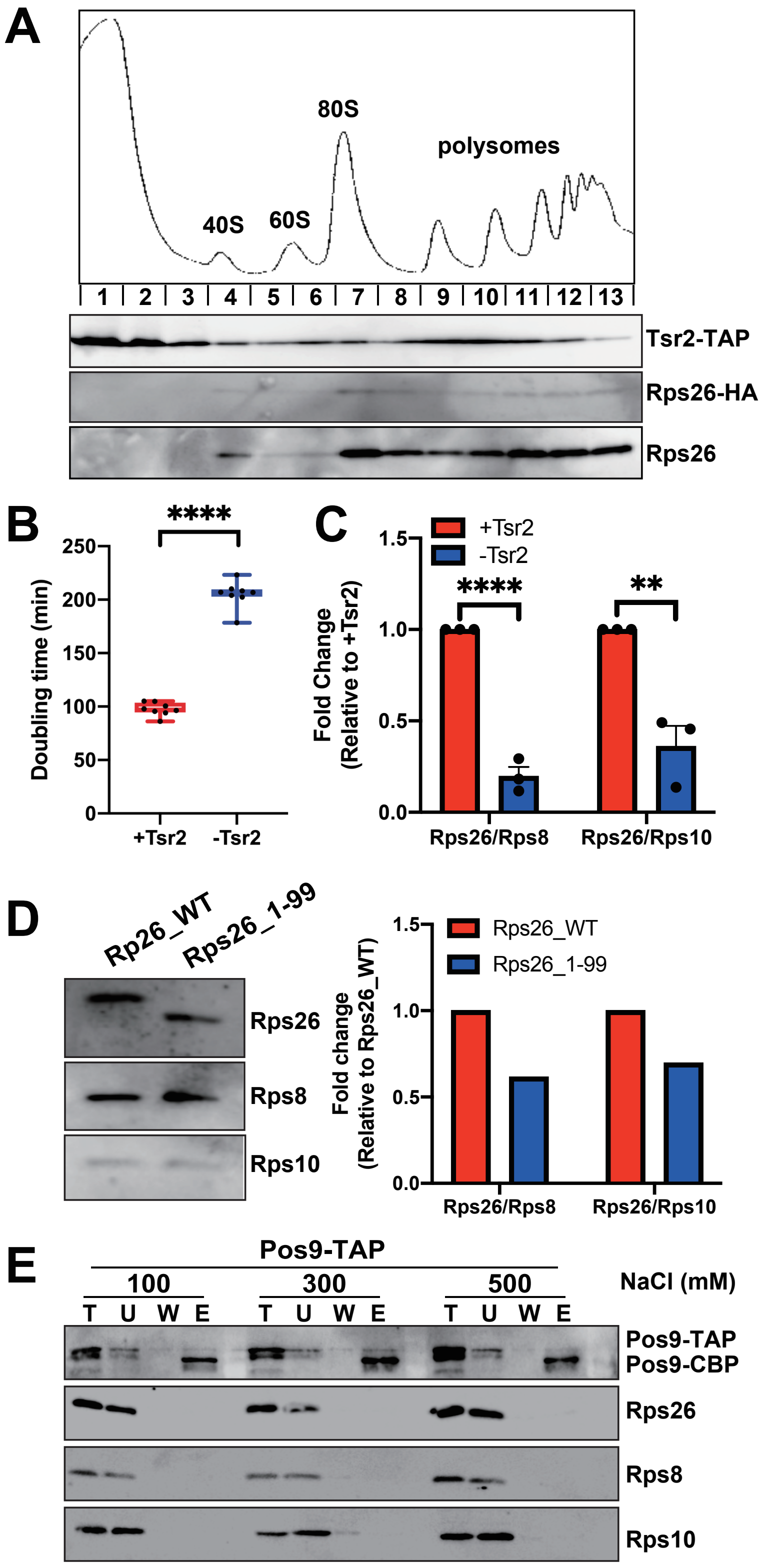
A
80S
polysomes
40S
60S
1 2 3 4 5 6 7 8 9 10 11 12 13
Tsr2-TAP
Rps26-HA
Rps26
B
****
250
200
150
100
50
0
Doubling time (min)
+Tsr2
-Tsr2
C
+Tsr2
-Tsr2
****
**
1.5
1.0
0.5
0.0
Fold Change
(Relative to +Tsr2)
Rps26/Rps8
Rps26/Rps10
D
Rp26_WT
Rps26_1-99
Rps26
Rps8
Rps10
Rps26_WT
Rps26_1-99
1.5
1.0
0.5
0.0
Fold change
(Relative to Rps26_WT)
Rps26/Rps8
Rps26/Rps10
E
Pos9-TAP
100
300
500
NaCl (mM)
T U W E
T U W E
T U W E
Pos9-TAP
Pos9-CBP
Rps26
Rps8
Rps10

Figure S3: Tsr2 is required for Rps26 release. (A) Rps26-HA is incorporated into 40S ribosomes and polysomes. Western analysis of fractions from a 10-50% sucrose gradient of yeast lysates from cells as in Figure 3, expressing plasmid-derived Rps26-HA in the background of genomic Rps26 and Tsr2-TAP. (B) Doubling times of Gal:Tsr2 yeast cells with and without plasmid-encoded Tsr2 grown in glucose. (C) Ribosomes from Gal:Tsr2 yeast cells grown for 18h in glucose have reduced amounts of Rps26, as analyzed by Western blotting relative to Rps8 and Rps10. Quantification of stress-free conditions in Figure 3D. (D) Western blot of ribosomes from cells where endogenous Rps26 is depleted and Rps26 wt or Rps26$^{1-99}$ is expressed from a plasmid, show that those from Rps26$^{1-99}$ cells have reduced amounts of Rps26. (E) Pos9-TAP does not co-purify ribosomes. IgG pulldown from cells containing TAP-tagged Pos9, analyzed using the TAP-antibody for Pos9, or for Rps26, Rps8 and Rps10. T, total lysate, U, unbound fraction, W, wash, E, elution.

FigureS4

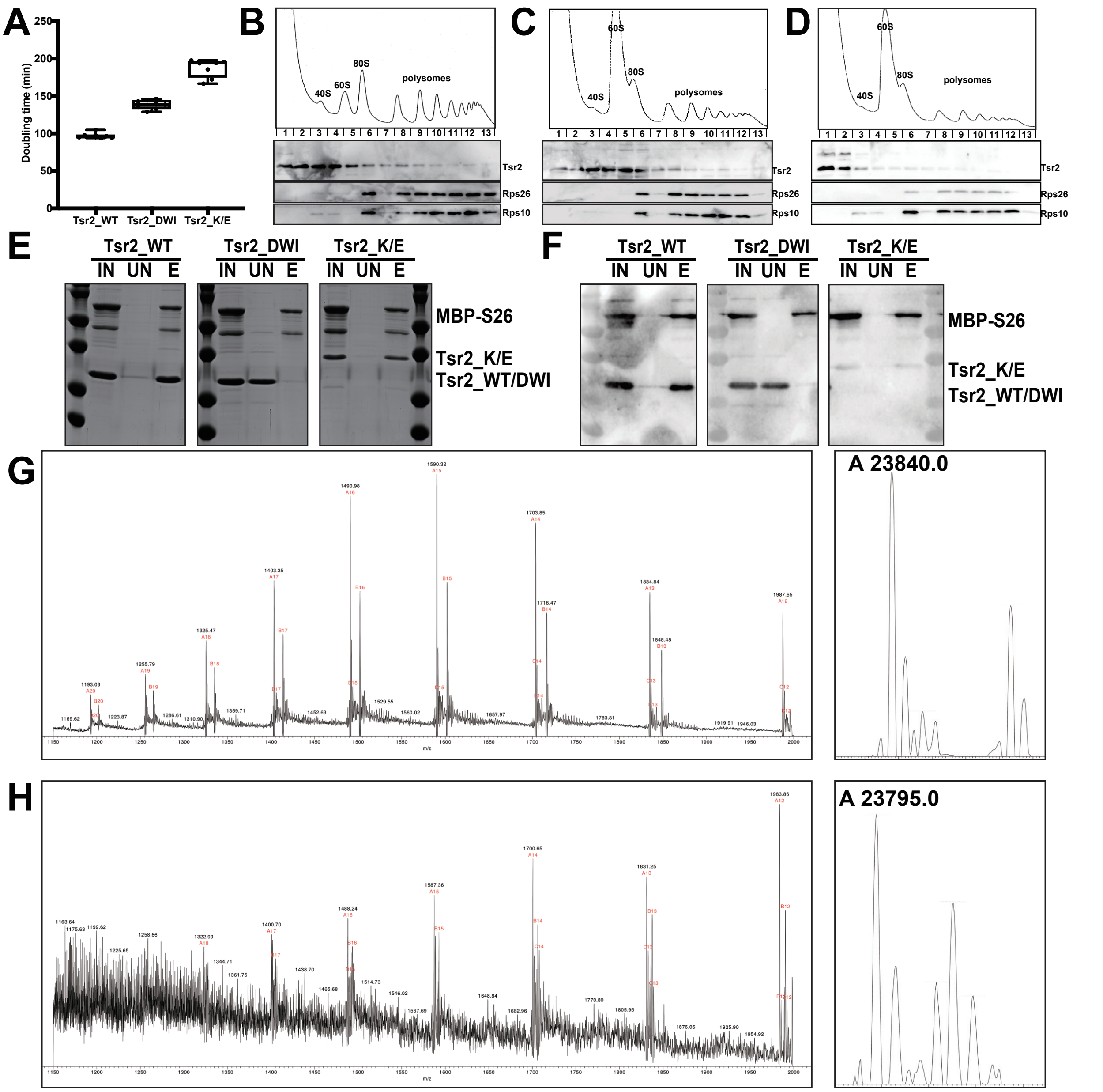

Figure S4: Release of Rps26 by Tsr2 under increased salt. (A) Changes in doubling time for YKK1109 cells (Gal:Tsr2) grown in glucose and supplemented with plasmids encoding Tsr2 variants. Data are averages from four biological replicates with two technical replicates each. 10-50% sucrose gradients of cell lysates from YKK1109 cells expressing plasmid-encoded Tsr2_WT (B) or Tsr2_DWI (C) or Tsr2_K/E (D). Shown below the absorbance profile at 254 nm are Western blots probing for Tsr2, Rps26, and Rps10. (E) Coomassie-stained SDS–PAGE of protein binding

assays with recombinant MBP-Rps26 and Tsr2 variants on amylose beads. IN, input; UN, unbound; E, elution. (F) Western blot probing for Tsr2 and Rps26 of the gel in panel (E). Because recombinant Tsr2_K/E runs with distinct electrophoretic mobility on the SDS-PAGE gel and reacts poorly to the Tsr2 antibody we confirmed its identity using ESI-MS mass-spectrometry, which gives the expected exact masses for both wild type Tsr2 (G) and Tsr2_K/E (H) variant. The right panel shows the deconvoluted spectra derived from multiply charged ions (Left).

FigureS5

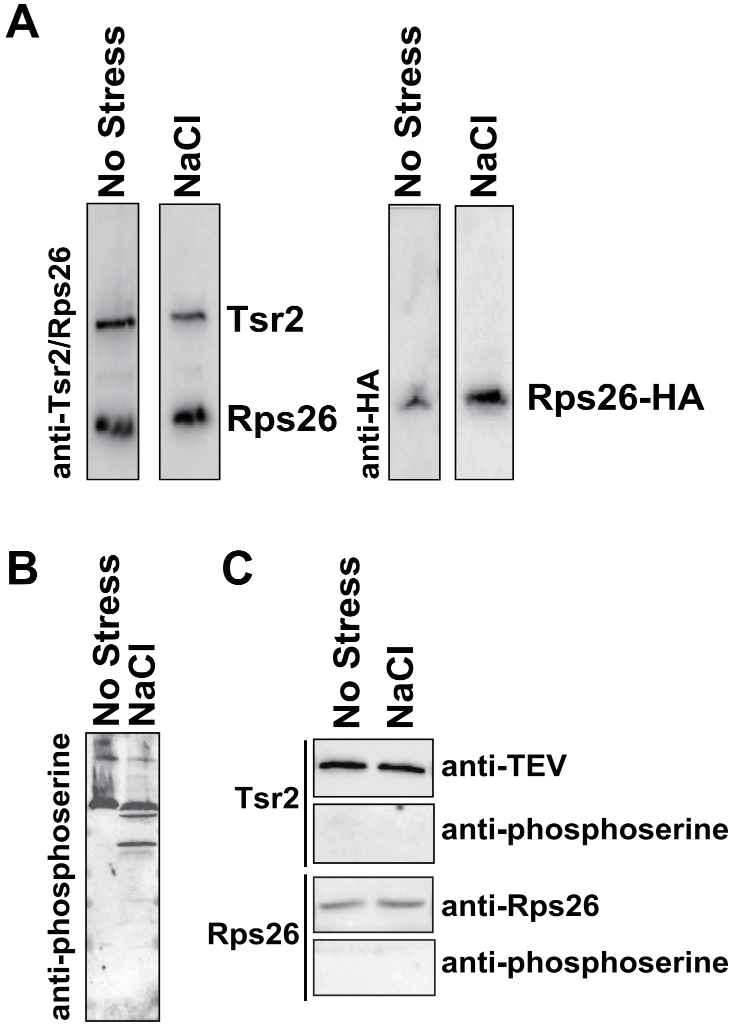

Figure S5: No post-translational modification observed in Rps26 and Tsr2 after stress. (A) Western blot of Rps26 and Rps26-HA from newly made and pre-existing ribosomes, respectively, co-isolated with TAP-tagged Tsr2 from cells that were or were not treated with 1 M NaCl. Elution samples were detected using primary antibodies of anti-Tsr2/Rps26 or anti-HA. The two samples were from the same gel. (B) Western blot of total cell lysate of cells treated with or without 1 M NaCl. The two samples were from the same gel. (C) Western blot of Tsr2-TAP purified from lysate in panel (B). An anti-phosphoserine antibody from Sigma (AB1603) was used to detect phosphorylated proteins in panel (B) and (C).

FigureS6

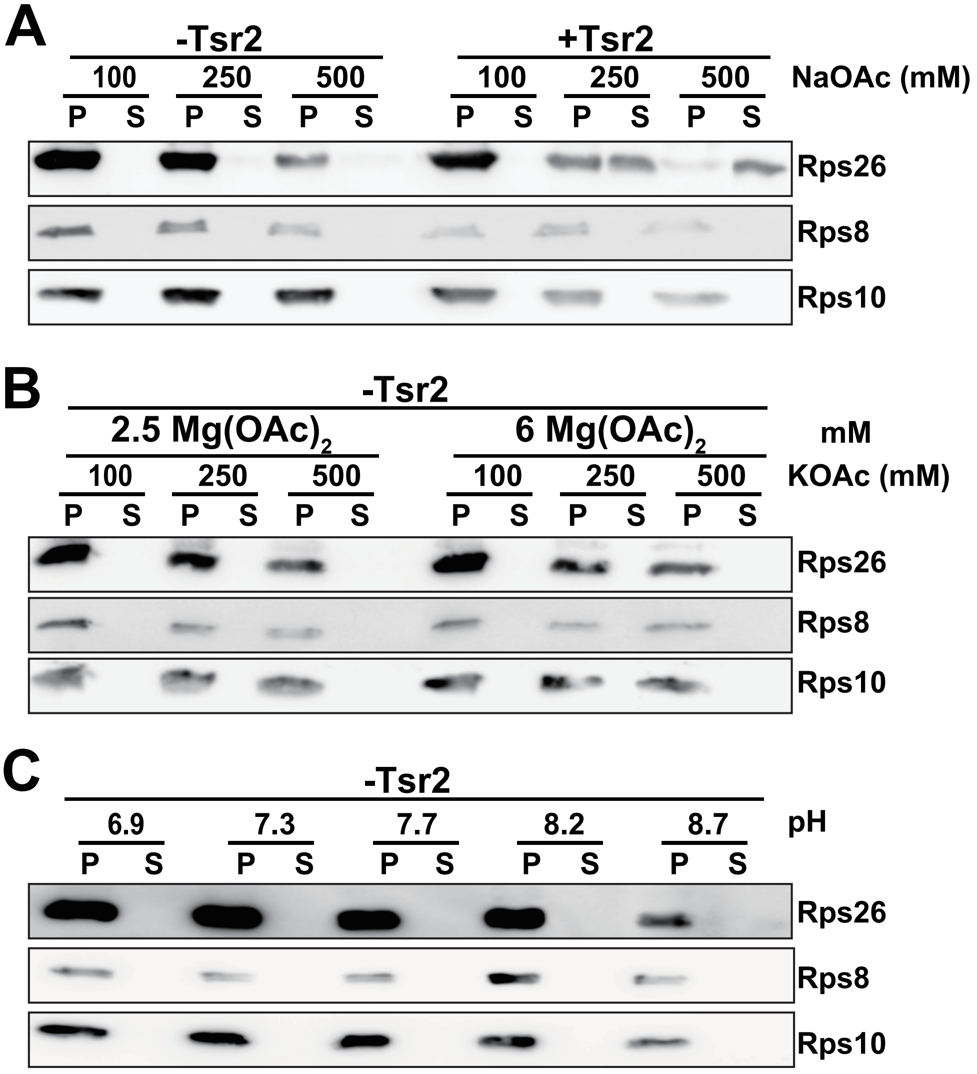

Figure S6: Release of Rps26 is Tsr2-dependent. (A) Tsr2 is required for Na^+ dependent release of Rps26. Release of Rps26, Rps8 and Rps10 was measured in presence and absence of Tsr2 at different Na^+ values by western blot. (B) Tsr2 is required for Mg^{2+} dependent release of Rps26. Release of Rps26, Rps8 and Rps10 was measured in absence of Tsr2 at different Mg^{2+} values by western blot. (C) Tsr2 is required for pH dependent release of Rps26. Release of Rps26, Rps8 and Rps10 was measured in absence of Tsr2 at different pH values by western blot.

FigureS7

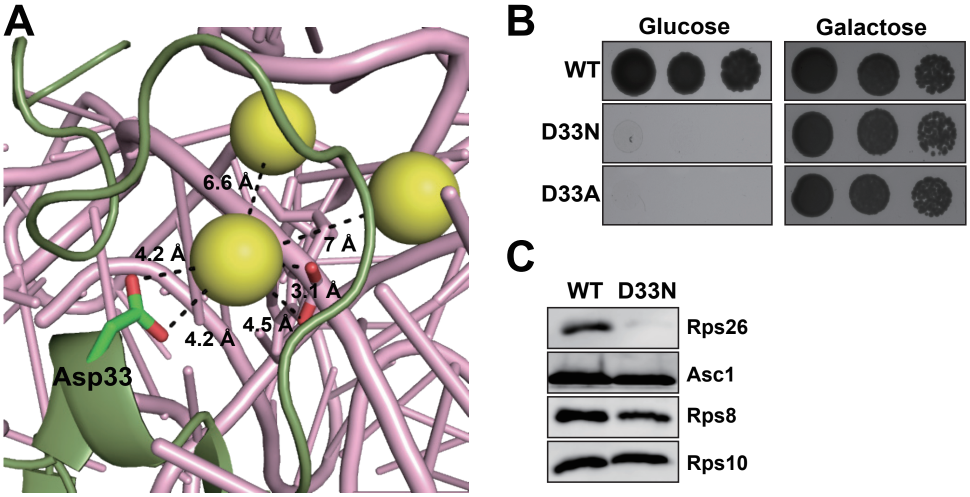

Figure S7. Effect of Asp33 mutation in Rps26. (A) Aspartate 33 in Rps26 binds a metal ion, displayed in yellow spheres (PDB 4V88). (B) Mutation of Asp33 mutation to asparagine or alanine leads to growth defects in yeast cells. Gal:Rps26A, Rps26Δ yeast cells containing plasmids encoding wt Rps26 or Rps26_D33N or Rps26_D33A were plated in 10 fold serial dilution either on glucose-containing media (to deplete endogenous Rps26) or on galactose media (to retain endogenous Rps26). (C) Occupancy of Rps26 in 40S subunits purified from cells expressing either Rps26_WT or Rps26_D33N.

Table S1: Yeast strains used in this work

Strain	Description	Background	Genotype	Reference
YKK200	WT	BY4741	*MATα his3Δ1 leu2Δ0 met15Δ0 ura3Δ0*	GE Dharmacon
YKK491	Gal::Rps26	BY4741	*MATα NatMX6::pGAL1-Rps26A Rps26B::KanMX6 his3Δ1 leu2Δ0 met15Δ0 ura3Δ0*	(*12*)
YKK493	Gal::Rps3	BY4741	*MATα KanMX6::pGAL1-Rps3 his3Δ1 leu2Δ0 met15Δ0 ura3Δ0*	(*12*)
YKK856	Tsr2-TAP	BY4741	*MATα NatMX6::Tsr2-TAP his3Δ1 leu2Δ0 met15Δ0 ura3Δ0*	GE Dharmacon
YKK1109	Gal-Tsr2	BY4741	*MATα NatMX6::pGAL1-Tsr2 his3Δ1 leu2Δ0 met15Δ0 ura3Δ0*	(*27*)
YKK95	Pos9-TAP	BY4741	*MATα NatMX6::Pos9-TAP his3Δ1 leu2Δ0 met15Δ0 ura3Δ0*	GE Dharmacon

Table S2: Plasmids used in this work

Plasmid	Description	Backbone	Reference
pKK3558	TEF::Rps26A	pRS416	(*12*)
pKK30832	TEF::Rps26A_D33N	pRS416	This work
pKK30831	TEF::Rps26A_D33A	pRS416	This work
pKK30528	Gal::Rps26A-HA	pRS426	This work
pKK30592	TEF::Rps26A-HA	pRS416	This work
pKK30562	TEF::Rps26A$^{1-99}$-HA	pRS416	This work
pKK30012	TET:: Rps3-TAP	pCM189	This work
pKK30221	TEF::Tsr2	pRS415	This work
pKK30935	TEF::Tsr2_K/E (K18E;K75E;K78E;K42E;K127E;K130; R140E;K142E;K143E;K145E;R146E)	pRS415	This work
pKK30570	TEF::Tsr2_DWI	pRS415	This work

Table S3: qPCR primers used in this work

Gene	Type	Sequence
Rps3	FW	GCTGTCACCATCATTGAACC
	RV	GCACTAGAATAGAAGAAATTATTG
Rps3-TAP	FW	GCTGTCACCATCATTGAACC
	RV	CAAGTGCCCCGGAGGATGAG
PGK1	FW	GCTGCTTTGCCAACCATCAA
	RV	GGCTTCAACTTCTGGACCGA
